# The complete chloroplast genome of *Lonicera oblata*, a critically endangered species endemic to North China

**DOI:** 10.1080/23802359.2019.1629344

**Published:** 2019-07-12

**Authors:** Yi-Xuan Zhu, Yuan-Mi Wu, Xue-Li Shen, Ling Tong, Xiao-Fei Xia, Xian-Yun Mu, Zhi-Xiang Zhang

**Affiliations:** a Laboratory of Systematic Evolution and Biogeography of Woody Plants, College of Nature Conservation, Beijing Forestry University, Beijing, P. R. China;; b Beijing Museum of Natural History, Beijing, P. R. China

**Keywords:** *Lonicera oblata*, endangered species, complete chloroplast genome, Illumina sequencing, North China

## Abstract

*Lonicera oblata*, a critically endangered species endemic to North China with about 30 wild individuals, has long been ignored for conservation since its publication because of little attention on its living situation. In this study, we characterized the complete chloroplast (cp) genome of *L. oblata*. The cp genome was 155,481 bp in length, included a large single-copy (LSC) region of 89,139 bp, a small single-copy (SSC) region of 18,676 bp, and two inverted repeat (IR) regions of 23,833 bp each. The genome contains 130 genes, including 85 protein-coding genes, 37 tRNA genes, and 8 rRNA genes. Phylogenetic position of *L. oblata* was also investigated based on cp genome phylogeny of *Lonicera* representatives. This study is valuable for molecular phylogenetic study and conservation of *Lonicera* and related taxa.

*Lonicera oblata*, a critically endangered deciduous shrub belongs to the family Caprifoliaceae, is endemic to northern China which only can be found at stony hillsides with an altitude of about 1000 m. However, this species is in critically endangered condition due to climate change, serious environmental degradation, human disturbance, and low propagation coefficient. At present, just about 30 wild living individuals are found sparsely distributed in Beijing City, Hebei and Shanxi Provinces in China. Although threatened by extinction, the conservation of *L. oblata* has been ignored for a long time. Genetic diversity is of great importance in conservation plan for endangered plants. In this study, we assembled the complete chloroplast genome of *L. oblata* and the annotated chloroplast genome sequence has been deposited in the GenBank with an Accession Number MH681655. This study will not only help to investigate population genetic diversity of *L. oblata*, but also contribute to the molecular phylogeny studies of *Lonicera* and Caprifoliaceae.

The fresh leaves of *L. oblata* were collected from Song Mountain, Beijing, China (N 115°43′44″–115°50′22″, E 40°29′9″–40°33′35″). Voucher specimen (collector and collection number: *Xian-Yun Mu 3877*) is deposited in the herbarium of Beijing Forestry University. Genomic DNA extraction and next-generation sequencing were performed with an Illumina Hiseq platform by Shanghai OE Biotech. Co., Ltd. (Shanghai, China). In total, 16.91 Gb of 150 bp clean reads were generated and used for chloroplast genome assembly through Geneious 11.1.4 software (Kearse et al. 2012) with *Lonicera japonica* (Kang et al. 2018) as a reference sequence. The assembled chloroplast genome was then annotated using the Plann (Huang and Cronk 2015). Eventually, annotations were verified by Geneious 11.1.4 software.

The complete chloroplast genome of *L. oblata* was 155,481 bp in length and had a typical quadripartite structure. The genome included a large single-copy (LSC) region of 89,139 bp, a small single-copy (SSC) region of 18,676 bp, and two inverted repeat (IR) regions of 23,833 bp each. Overall, the GC content was 38.4%. A total number of 130 genes in the *L. oblata* chloroplast genome was identified, including 85 protein-coding genes, 37 tRNA genes, and 8 rRNA genes.

Phylogenetic analysis was conducted using 14 species from *Lonicera*, and one outgroup specie from *Triosteum* (Figure 1). A total of 15 complete chloroplast genomes were aligned using MAFFT (Katoh et al. 2017) and adjusted manually. The maximum likelihood (ML) tree was constructed with IQ-TREE software (Kalyaanamoorthy et al. 2017; Hoang et al. 2018). The result accord with the classification of Subgen. *Chamaecerasus* and Subgen. *Lonicera* in traditional classification system, but the phylogenetic relationship intra Subgen. *Chamaecerasus* needs further investigation.

**Figure 1.**
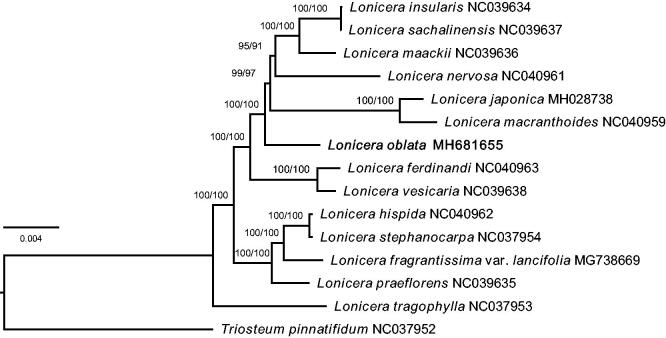
Maximum likelihood tree of 15 Caprifoliaceae chloroplast genomes. The position of *L. oblata* is shown in bold. Numbers above nodes represent values of the Ultrafast bootstrap and SH-aLRT which are generated from IQ-TREE.
